# Risk of Menstrual Dysfunction, Low Energy Availability, Eating Disorders and Injury in the First All-Female UK Military Team Rowing 3000 Miles Across the Atlantic

**DOI:** 10.3390/sports14060256

**Published:** 2026-06-22

**Authors:** Solène Chaléat, David Baud, Helton De Sa Souza, Imogen O’Brien, Rebecca Glover, George Morris, Kelly Kaulback, Volker Scheer

**Affiliations:** 1 Materno-Fetal and Obstetrics Research Unit, Department Woman-Mother-Child, Lausanne University Hospital (CHUV) and University of Lausanne (UNIL), 1011 Lausanne, Switzerland; solene.chaleat@chuv.ch; 2 Department of Physical Education, Universidade Federal de Vicosa, Vicosa 36570-900, Brazil; 3 Psychobiology and Exercise Laboratory, Universidade Federal de Vicosa, Vicosa 36570-900, Brazil; 4 PCRF Aldershot, McNaughton House, Rawlinson Road, Aldershot, Hampshire GU11 2LQ, UK; 5 Medical Training and Support Unit, Robertson House, Slim Road, Camberley GU11 2LQ, UK; 6 School of Sport, Exercise and Applied Science, Faculty of Sport, Technology, and Health Sciences, St Mary’s University, Twickenham, London TW1 4SX, UK; 7 School of Allied Health and Life Sciences, Faculty of Sport, Technology, and Health Sciences, St Mary’s University, Twickenham, London TW1 4SX, UK; 8 Ultra Sports Science Foundation, 69310 Pierre-Benite, France; volkerscheer@yahoo.com; 9 Royal Air Force Honington, Regional Rehabilitation Unit Colchester, Honington Clinic, Bury St. Edmunds, Suffolk IP31 1EE, UK

**Keywords:** menstrual health, energy deficiency, ultra-endurance exercise, female athletes, transatlantic rowing

## Abstract

Data on menstrual health, energy availability, and injury risk in women undertaking extreme ultra-endurance expeditions remain limited. We conducted a prospective cohort study of the first all-female UK military team competing in a 3000-mile transatlantic rowing race, aiming to characterize menstrual function, low energy availability (LEA) risk, eating disorder (ED) risk, and injury profiles. Four female British Army personnel completed the 46-day race. Menstrual symptoms, injuries, and illnesses were recorded daily, while reproductive, inflammatory, biochemical, and hematological markers were assessed before and after the race. LEA and ED risk were evaluated using the Low Energy Availability in Females Questionnaire and Brief Eating Disorder in Athletes Questionnaire, respectively. Analyses were primarily descriptive. Three athletes experienced amenorrhea during the expedition, including one with previously regular cycles. The fourth reported intermittent abnormal bleeding associated with injury and illness and screened positive for LEA risk before and after the race. Another athlete screened positive for ED risk at both time points. Most biomarkers remained stable post-race, whereas reproductive hormones showed consistent reductions in follicle-stimulating hormone and luteinizing hormone in all four participants, alongside increased oestradiol. These findings, based on a sample of four athletes, suggest that menstrual function may be sensitive to sustained physiological stress in extreme ultra-endurance settings, and support prospective monitoring in female ultra-endurance, military, and expeditionary populations.

## 1. Introduction

Despite the proliferating popularity of ultra-endurance sports, women remain underrepresented in both participation and empirical research, particularly in extreme disciplines such as transoceanic rowing [1,2,3]. The existing literature within the ultra-endurance domain is predominantly derived from studies conducted in male athletes, with findings often extrapolated to female populations without adequate consideration of sex-specific physiological nuances [4]. Consequently, female-specific evidence remains limited, despite the unique health challenges women face regarding energy availability (EA), menstrual function, and injury risk, which may be potentiated in ultra-endurance settings [5,6,7]. Understanding these risks has direct practical relevance for monitoring strategies, injury prevention, and the health and operational readiness of female military and expeditionary personnel.

Low energy availability (LEA), defined as insufficient energy intake to support simultaneously exercise energy expenditure and normal physiological function [8,9], is a primary driver of menstrual dysfunction and impaired bone health in female athletes [8,10]. LEA triggers metabolic and hormonal adaptations [8,9] associated with increased injury risk, impaired recovery, and menstrual disturbance [6,9] ranging from luteal phase defects to amenorrhea [10,11]. Athletes at risk for LEA demonstrate a substantially higher likelihood of musculoskeletal injuries compared with those with adequate energy availability [9,12]. Importantly, LEA can occur inadvertently due to elevated exercise energy expenditure alone, even in the absence of disordered eating [13].

The Female Athlete Triad describes the interrelationship between energy availability, menstrual function, and bone health [8,10]. Relative Energy Deficiency in Sport (RED-S) extends this model to encompass broader systemic consequences affecting metabolic, immune, cardiovascular, and psychological health in both sexes [8,9].

However, data characterising menstrual health, energy availability and injury patterns in women undertaking prolonged ultra-endurance expeditions remain sparse, particularly in disciplines such as ocean rowing. Ocean rowing is established as discipline demanding high physical and technical capacity [1]. Presently, ocean rowing represents one of the most extreme forms of ultra-endurance exercise, requiring athletes to row continuously for weeks across thousands of miles of open water under conditions of cumulative fatigue, sleep deprivation, repeated mechanical loading, and sustained energy demands, all of which exacerbate the risk of illness and injury [3,14]. Since the inaugural ocean rowing crossing was accomplished in 1869, and it has since grown to around 200 recorded attempts per year [2]. This study aimed to characterise menstrual health, energy availability and injury patterns in the first all-female UK military team to row 3000 miles across the Atlantic Ocean.

## 2. Material and Methods

### 2.1. Study Design and Participants

The characteristics of the race and the participants have been previously detailed [1,2]. In summary, active-duty female British Army personnel were recruited via an open invitation. Prospective candidates underwent a multi-stage selection process comprising physical, cognitive and leadership assessments, followed by an intensive training phase. The final expedition team comprised four women, constituting the first all-female British Army crew to undertake this Atlantic crossing.

None of the rowers had prior ocean rowing experience. Preparation commenced approximately two years prior to departure. The training regimen incorporated endurance conditioning and strength training sessions conducted multiple times per week, with boat-based technical preparation introduced during the final months before the race (≈140 h on water).

### 2.2. Expedition Characteristics

The team competed in the 2024–2025 Atlantic edition of the World’s Toughest Row, covering ~3000 miles from La Gomera (Canary Islands) to Antigua. They completed the crossing in 46 days and 55 min (concluding in January 2025), securing 18th place overall and second in the all-female four-person category. Environmental conditions fluctuated from nocturnal lows of ~10 °C to diurnal peaks of ~40 °C, exhibiting high humidity and repeated exposure to high-velocity winds and rough seas. Rowing was generally organised in alternating two-hour shifts in pairs of two throughout the 24 h cycle. Rest periods were allocated for nutritional intake, maintenance, navigation checks and sleep. Sleep was primarily obtained during night-time breaks in confined cabins, without pharmacological or mechanical aids. Daytime napping was seldom viable due to operational demands.

### 2.3. Biomonitoring and Psychometric Assessments

Venous blood samples were collected at three distinct time points: at baseline (pre-race), immediately post-race and 7 days post-race to evaluate physiological responses to the race. Laboratory analyses included a complete blood count with differential, iron status markers (including serum ferritin), inflammatory markers (erythrocyte sedimentation rate and C-reactive protein), and an extensive metabolic and biochemical panel. This panel encompassed electrolytes, renal and liver function tests, lipid profile, glucose metabolism markers, vitamin D status, vitamin B12, folate, and cortisol. Furthermore, muscle damage markers (creatine kinase), thyroid function, and a comprehensive reproductive endocrine profile were assessed, including follicle-stimulating hormone (FSH), luteinizing hormone (LH), œstradiol, sex hormone-binding globulin (SHBG), free androgen index (FAI), progesterone, testosterone, and prolactin. Timing of blood sampling was not standardised relative to circadian rhythm or menstrual cycle phase. Given the small sample size and the strong dependence of reproductive hormone concentrations on menstrual cycle phase and hormonal contraceptive status, reproductive hormone results were interpreted at the individual level only. No group mean hormonal profile was calculated for LH, FSH, œstradiol, progesterone, or other reproductive hormones. These measurements were considered exploratory and hypothesis-generating.

### 2.4. Clinical Surveillance

Menstrual health was monitored daily throughout the race via self-report. For the purposes of this study, amenorrhoea was defined as the absence of menstrual bleeding throughout the entire 46-day race period. Abnormal bleeding was defined as any bleeding episode occurring outside the participant’s normal menstrual pattern, including variation in timing, duration, or intensity. Participants recorded the presence and typology of menstrual symptoms, further indicating the perceived impact on athletic performance.

Similarly, illness and injury surveillance was conducted daily during the race. Injury was defined as any self-reported musculoskeletal complaint (e.g., hand, finger, toe or foot soft tissue injury, elbow, shoulder, groin or back pain) occurring during the race. Illness was defined as any self-reported non-musculoskeletal condition (e.g., gastrointestinal symptoms, dermatological condition, seasickness). Participants reported any injury or illness, specifying symptomatology and anatomical location. No formal severity grading scale was used; however, participants were asked to report the perceived impact of each event on performance, categorised as none, minor, or major.

Responses were recorded daily on participants’ electronic devices and/or laminated paper records that were photographed daily. Each participant noted the date and time of completion. Data were later transcribed by the researchers for data analysis, as described previously [1].

### 2.5. Energy Availability and Eating Behavior Assessments

Disordered eating (DE) and Risk of low energy availability (LEA) were evaluated pre- and post-race using two validated instruments: the Brief Eating Disorder in Athletes Questionnaire (BEDA-Q) and the Low Energy Availability in Females Questionnaire (LEAF-Q), respectively. The BEDA-Q [15] is a validated self-report screening tool designed to identify eating disorder risk in female athletes. The questionnaire assesses key domains including drive for thinness, body dissatisfaction, perfectionism and dieting behaviors. A BEDA-Q score ≥ 0.27 was used as the cutoff to identify athletes at risk of an eating disorder [15,16].

The LEAF-Q [17] is a validated 25-item screening instrument designed to identify female athletes at risk of LEA, and consequently, at risk of developing Relative Energy Deficiency in Sport (RED-S). The questionnaire assesses three domains: injuries and illnesses, gastrointestinal function, and menstrual function. A total score of ≥8 was used as the cutoff to identify athletes at risk for low energy availability and its associated physiological consequences [9,17].

### 2.6. Analyses, Study Approval and Ethical Considerations

Due to the small sample size of four participants, analyses were primarily descriptive, with individual-level reporting of clinical events, questionnaire scores, and biomarker changes before and after the race. Inferential statistics were not performed, as the sample size precludes meaningful hypothesis testing. This case-series approach was considered appropriate given the exploratory nature of the study and the logistical constraints inherent to transoceanic ultra-endurance expeditions. Descriptive statistics included means, medians and ranges for continuous variables, and individual-level trajectories were summarised graphically where appropriate.

Prior to initial testing, all participants received detailed information about the study protocol and gave their written informed consent. The Faculty of Sport, Technology and Health Sciences Ethics Committee at St Mary’s University, London, granted ethical approval (reference number SMU_ETHICS_2024-25_745) [18]. The research adhered to the principles outlined in the Declaration of Helsinki and followed the STROBE (Strengthening The Reporting of Observational studies in Epidemiology) reporting guidelines for cohort studies [19].

## 3. Results

**Figure 1:**
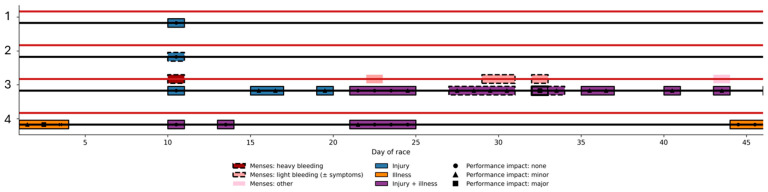
Integrated rowing timelines of menstrual function (red line) and injury or illness (black line) for the four participants (Rowers 1, 2, 3 and 4) across the 46-day Atlantic crossing.

The timelines of menstrual symptoms, injuries and illnesses reported by the four participants (Rowers 1, 2, 3 and 4) during the crossing are illustrated in Figure 1.

### 3.1. Menstrual Function

Three of the four participants (Rowers 1, 2 and 4) experienced amenorrhea throughout the race, with no menstrual bleeding reported during the expedition. Two of these (Rowers 2 and 4) used an intrauterine device and had reported occasional absent bleeding prior to the expedition. Rower 1 had previously regular, normal menstrual cycles but had noticed exercise-associated reductions in menstrual bleeding before the race. During the 46-day race, she experienced complete amenorrhea. The fourth participant (Rower 3) reported menstrual cycle disturbances, characterised by intermittent abnormal bleeding on 7 days over the 46-day crossing, ranging from light to heavy intensity. She had indicated a prior history of menstrual irregularities, including cycle interruptions lasting three months or longer (excluding pregnancy), with her last menstrual cycle occurring approximately 3 months before the race.

### 3.2. Injury and Illness

Reported injuries were minor musculoskeletal complaints affecting the hands and fingers (soft tissue irritation and chafing), toes and feet (pressure-related lesions), as well as elbow, shoulder, groin or back pain. Reported illnesses included seasickness during the early phase of the crossing, transient gastrointestinal symptoms, fatigue, or dermatological conditions. Details of the frequency and duration of each event for each participant are presented in Figure 1. All four participants reported at least one injury or illness episode during the race, with multiple events occurring in two rowers (Rowers 3 and 4). Moreover, all participants sustained an injury on day 10 of the crossing. Most events were self-limited and did not result in self-reported performance impairment. Rowers 3 and 4 experienced the highest number of events. In Rower 3, these included dermatological lesions affecting the hands and fingers, as well as groin and back pain. In Rower 4, events included toes and foot lesions, elbow pain, gastrointestinal symptoms, and seasickness. The participant with the highest cumulative burden of injury and/or illness (Rower 3), accounting for 19 days affected over the 46-day race, was also the only rower to report abnormal menstrual bleeding during the crossing. In this athlete, bleeding episodes were reported only on days when both musculoskeletal injury (hand and finger lesions, as well as groin or back pain) and illness (dermatological condition and fatigue) were also recorded. On these days, self-reported performance impairment, described as either minor or major, was also noted. No bleeding episodes were reported on days without injury or illness.

### 3.3. Energy Availability and Eating Disorder Risk

**Figure 2:**
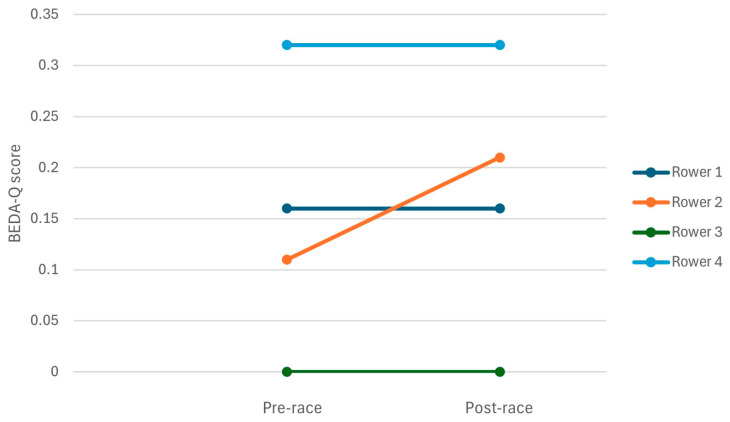
Individual BEDA-Q scores of participants.

Results of the BEDA-Q questionnaires before and after the race are shown in Figure 2. Individual BEDA-Q scores showed minimal variation between pre- and post-race assessments. One out of four athletes (Rower 4) scored above the validated cutoff of 0.27 at both time points, whilst the remaining three scored below the cutoff at both time points.

**Figure 3:**
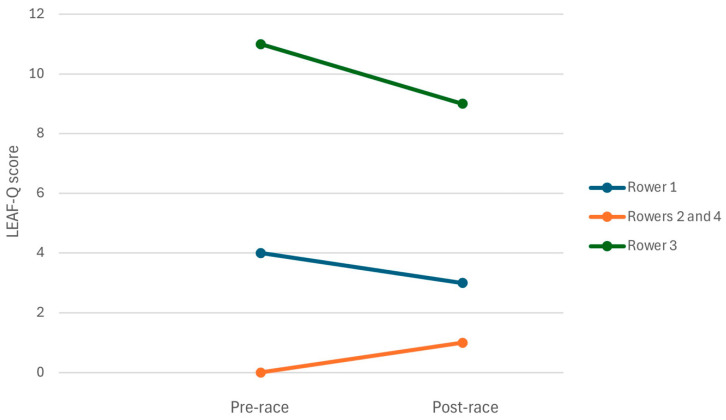
Individual LEAF-Q scores of participants.

Results of the LEAF-Q questionnaires before and after the race are shown in Figure 3. One out of four participants (Rower 3) scored above the validated cutoff of 8 both before and after the race, whilst the remaining three scored below the cutoff at both time points. This was the same athlete who reported menstrual disturbances during the race, including intermittent abnormal bleeding episodes.

### 3.4. Blood Biomarkers

Individual biomarker values and percentage changes are detailed in Table S1 (Supplementary Material). Overall, blood biomarkers remained stable following the race, with no major differences observed between pre- and post-race measurements. Creatine kinase (CK), a sensitive marker of skeletal muscle damage, decreased post-race in all four athletes (mean/median: 279/164 U/L [range: 98–575] pre-race vs. 72/69 U/L [range: 48–104] post-race), and remained within the normal range (<200 U/L). Markers of systemic inflammation (CRP and ESR), hematological parameters, and renal function markers (creatinine and eGFR) remained within physiological ranges across all participants.

Reproductive hormone values are presented descriptively at the individual level in Table S1. Because hormone concentrations depend strongly on menstrual cycle phase and hormonal contraceptive status, no group mean hormonal profile was calculated. At the individual level, post-race FSH and LH values were lower than pre-race values, whilst œstradiol values were higher post-race, consistently across all four participants.

## 4. Discussion

### 4.1. Main Findings

This study aimed to characterise menstrual health, energy availability risk, eating disorder risk, and injury profiles in women undertaking an extreme ultra-endurance expedition. During the 46-day Atlantic crossing, three of four athletes experienced amenorrhea, while the remaining rower reported abnormal bleeding episodes that occurred on days with concurrent injury or illness. One participant screened positive for eating disorder risk (BEDA-Q) and another for low energy availability (LEAF-Q), the latter being the same athlete who exhibited menstrual disturbances during the race. Although most blood biomarkers remained stable, reproductive hormones showed a pattern of reduced FSH and LH with increased œstradiol post-race in all four athletes, suggesting possible transient modulation of the hypothalamic–pituitary–ovarian axis under extreme ultra-endurance stress.

### 4.2. Blood Biomarker Responses to Prolonged Ultra-Endurance Rowing

It is well documented that ultra-endurance exercise can lead to acute inflammatory, hematological [3], and renal changes [20,21]; however, this was not observed in our results. Our findings suggest that, although modulation of reproductive hormones occurred, classical markers of muscle damage, inflammation, and renal function remained largely stable, despite the prolonged effort of rowing across the Atlantic Ocean.

CK levels decreased in all four athletes post-race, which may suggest that the intense strength and conditioning training performed prior to the expedition elicited greater acute muscle damage than the continuous, rhythmic rowing motion during the race. Systemic inflammation markers (CRP and ESR) remained low or only mildly elevated despite prolonged sleep deprivation, environmental stress, and repetitive mechanical loading inherent to rowing. Together, these observations suggest that the four athletes tolerated the musculoskeletal and inflammatory demands of the expedition well, possibly reflecting adaptation to continued physical effort.

### 4.3. Menstrual Health

Female athletes engaging in intense exercise or competition commonly exhibit central suppression of the hypothalamic–pituitary–ovarian (HPO) axis, characterised by suppressed gonadotropin-releasing hormone (GnRH) pulsatility, which leads to decreased follicle-stimulating hormone (FSH) and luteinizing hormone (LH) secretion, ultimately resulting in altered ovarian œstrogen production [5,22]. This pattern is characteristic of functional hypothalamic amenorrhea and is well documented in athletes participating in sports emphasizing leanness or high energy expenditure, such as endurance disciplines [5,23].

In the present study, three of four athletes experienced amenorrhea during the expedition. In one participant with previously regular cycles, the onset of complete amenorrhea during the race may suggest an exercise-associated suppression of the HPO axis under extreme ultra-endurance stress [5,22,23]. However, two participants were using intrauterine devices and had reported a prior history of absent bleeding, while the third reported previous exercise-associated menstrual disturbances. Pre-existing menstrual irregularities and hormonal contraceptive use are important confounders that preclude definitive conclusions regarding race-induced menstrual dysfunction. Caution is therefore warranted when interpreting the prevalence of amenorrhea in this cohort as a race-induced phenomenon.

Interestingly, our results showed an unexpected elevation in œstradiol levels in the days following intense competition, despite concomitant reductions in FSH and LH post-race. This apparent dissociation may reflect peripheral mechanisms independent of central gonadotropin drive; however, the underlying mechanisms cannot be determined from the present data. Possible explanation proposed in the literature include stress-induced hormonal coupling independent of the HPO axis [24] and enhanced peripheral aromatase activity in response to acute physical and psychological stress [25,26], though these remain speculative in the context of this small cohort.

### 4.4. Energy Availability and Eating Disorder Risk

One participant screened above the validated cutoff for low energy availability risk (LEAF-Q) both before and after the expedition, suggesting a pre-existing chronic vulnerability prior to the race rather than a race-induced change. The remaining three participants scored below the threshold at both time points. Interestingly, the LEAF-Q score of the participant above the cutoff decreased post-race, while remaining above the risk threshold. Notably, this was the same athlete who reported menstrual disturbances during the race and accumulated the highest injury and illness burden.

The LEAF-Q is designed to identify athletes at risk of low energy availability and components of Relative Energy Deficiency in Sport (RED-S), including menstrual dysfunction and increased injury susceptibility [19]. In the present study, the LEAF-Q scores did not indicate an increase in questionnaire-defined risk of low energy availability across the cohort. These findings may appear inconsistent with the substantial energetic stress of the expedition and the observed body mass loss. This may reflect the limited sensitivity of questionnaire-based tools such as the LEAF-Q to detect short-term changes in energy availability during extreme ultra-endurance events.

Only one participant screened positive for eating disorder risk (BEDA-Q), and this risk profile remained stable pre- and post-race, suggesting a chronic vulnerability rather than a transient race-induced behavioural change.

### 4.5. Injury-Illness Burden and Its Interaction with Menstrual Function

All participants experienced at least one injury or illness episode; however, the cumulative burden varied substantially. The athlete with the highest injury and illness load was also the only participant who screened above the LEAF-Q cutoff both before and after the race and the only one reporting abnormal bleeding during the crossing. Strikingly, bleeding episodes in this participant occurred exclusively on days when both injury and illness were concurrently recorded and were frequently associated with perceived performance impairment. Although causality cannot be established, this temporal clustering raises the hypothesis that periods of increased physiological strain may exacerbate menstrual instability in athletes already vulnerable to chronic low energy availability. Conversely, it is also conceivable that abnormal bleeding and associated hormonal fluctuations may have influenced symptom perception, pain sensitivity, or injury experience, contributing to the reported performance impairment. These observations are based on a single participant and should therefore be considered hypothesis-generating only.

Elevated LEAF-Q scores have been associated with increased injury risk in athletic populations [12]. The present observations are consistent with this framework, suggesting that menstrual disturbance, chronic low energy availability risk, and susceptibility to injury may represent interrelated manifestations of underlying physiological vulnerability in extreme endurance contexts, rather than race-induced effects.

### 4.6. Interaction with Sleep, Fatigue, Mood and Body Mass Loss in the Same Cohort

In the previously published study conducted in the same participants during the same 46-day Atlantic crossing [1], mood fluctuations (particularly tension and fatigue), severely restricted sleep (3.8 h per day), and significant body mass loss (15.5%) were documented. These findings provide important contextual insight into the menstrual disturbances observed in the present study.

The marked body mass loss suggests sustained negative energy balance throughout the expedition. Prolonged low energy availability (LEA) is a well-established trigger of functional hypothalamic suppression, mediated by reduced GnRH pulsatility and downstream reductions in FSH and LH secretion [10,11], and is physiologically consistent with the amenorrhea observed in three of four participants.

Chronic sleep restriction may have further contributed to menstrual dysfunction, as sleep deprivation is known to disrupt GnRH pulsatility and interfere with ovarian steroidogenesis [27,28].

Meticulous preparation and high resilience among participants may partly explain the relative stability of most systemic biomarkers despite weight loss, profound energy deficit and sleep restriction. Nevertheless, reproductive function appeared to be sensitive to the cumulative stress load in this cohort, raising the hypothesis that menstrual health may serve as an early and sensitive biomarker of physiological strain in extreme ultra-endurance environments [7,29].

These findings make several contributions to sports science. First, they demonstrate the feasibility of prospective menstrual health monitoring during extreme expeditionary settings using simple daily self-report tools. Second, they suggest that menstrual changes may serve as early warning signs of physiological overload in female athletes, warranting attention in future monitoring protocols.

## 5. Limitations

Reproductive hormones were assessed at only two time points (pre- and post-race) and from single blood samples. Furthermore, timing of blood sampling was not standardised relative to circadian rhythm or menstrual cycle phase. Given that gonadotropins and ovarian steroids exhibit marked circadian and cycle-phase variability, this represents an important limitation for the interpretation of reproductive hormone values. Ideally, sampling would be standardised to the same time of day and menstrual cycle phase for each participant; however, this was not feasible in the context of a prolonged ultra-endurance expedition where menstrual cycles were irregular or suppressed in several participants.

A further limitation concerns the heterogeneous hormonal environment of the participants. Two athletes were using hormone-releasing intrauterine devices and reported a prior history of absent bleeding, while a third had pre-existing exercise-associated menstrual disturbances. This heterogeneity precludes any group-level interpretation of reproductive hormone data and limits the ability to attribute menstrual changes observed during the race to the expedition itself. Taken together with the small sample size of four participants, these factors substantially constrain the generalisability of the findings and the conclusions that can be drawn regarding race-induced menstrual dysfunction.

Furthermore, menstrual symptoms, injuries, and illnesses were self-reported daily, introducing potential perception bias. Under conditions of extreme physical fatigue, sleep deprivation, and psychological stress, participants may have under- or overestimated symptom severity. Additionally, social desirability bias may have led participants to minimise symptoms to avoid concerns about their performance or team contribution. The recording of date and time of completion on each form partially mitigates recall bias. Finally, this study included a small sample of four female rowers, so the findings cannot be generalised to a wider group of female ultra-endurance athletes. However, given the extreme and logistically complex nature of transoceanic ultra-endurance expeditions, assembling larger cohorts is inherently challenging. Future studies including more participants would strengthen the external validity of these findings and allow for more robust conclusions.

## 6. Conclusions

These results add to the limited data on female physiology in extreme endurance environments and underscore the sensitivity of menstrual function to sustained physiological stress. Despite overall systemic adaptation, reproductive parameters appeared particularly responsive to the demands of the crossing.

Prospective monitoring of menstrual function and energy availability in female ultra-endurance, military, and expeditionary cohorts may help identify early signs of physiological strain and inform strategies to preserve long-term health and performance.

## Data Availability

The data presented in this study are included in the article and Supplementary Material. Further inquiries can be directed to the corresponding author.

## Supplementary Materials

The following supporting information can be downloaded at: https://www.mdpi.com/article/10.3390/sports14060256/s1, Table S1: Biomarker values and percent change in each of the four participants.
