# Supplementary material for: Risk of Menstrual Dysfunction, Low Energy Availability, Eating Disorders and Injury in the First All-Female UK Military Team Rowing 3000 Miles Across the Atlantic
Source: Sports (Basel). 2026 Jun 22;14(6):256. doi: 10.3390/sports14060256 (PMC13306676; doi:10.3390/sports14060256)
Supplement: Supplementary file 1 [file sports-14-00256-s001.zip › sports-4330555-supplementary.pdf]

Table S1. Biomarker values and percent change in each of the four participants.

Parameter	Reference	Pre-race					Post-race					% difference of means	Pre-race value / post-race value	Percentage difference for each			
		Rower 1	Rower 2	Rower 3	Rower 4	MEAN	Rower 1	Rower 2	Rower 3	Rower 4	MEAN			Rower 1	Rower 2	Rower 3	Rower 4
**FBC**																	
Total white cell count 10*9/L	*4-11 U*		6.1	8.2	3.2	5.83	4.7	7.5	6	4.6	5.7	**0.02**	**1.02**		-0.23	0.27	-0.44
Haemoglobin g/L (Hb)	*115-165 U*		136	149	102	129	131	129	143	133	134	**-0.04**	**0.96**		0.05	0.04	-0.3
Platelet count 10*9/L	*150-450 U*		398	194	368	320	342	332	177	322	293.3	**0.08**	**1.09**		0.17	0.09	0.13
Red blood cell count (RBC) 10*12/L	*3.5-5-5 U*		4.48	4.95	3.97	4.47	4.46	4.32	4.5	3.97	4.31	**0.03**	**1.04**		0.04	0.09	-
Haematocrit	*0.37-0.47U*		0.4	0.49	0.32	0.4	0.4	0.39	0.452	0.39	0.41	**-0.01**	**0.99**		0.03	0.08	-0.22
Mean corpuscular volume (MCV) fl	*75-105 U*		89	99	80.3	89.43	90.2	91	101	99	95.3	**-0.07**	**0.94**		-0.02	-0.02	-0.23
Mean corpuscular haemoglobin (MCH) pg	*26-35 U*		30.4	30.2	25.7	28.77	29.3	29.8	31.7	33.4	31.05	**-0.08**	**0.93**		0.02	-0.05	-0.3
Mean corpuscular Hb concentration g/L	*290-350 U*		343	305	320	322.7		328	315	338	327	**-0.01**	**0.99**		0.04	-0.03	-0.06
Red cell distributin width %	*11-15 U*		12.8	13	17.9	14.57	12.8	13.1	13.6	13.9	13.35	**0.08**	**1.09**		-0.02	-0.05	0.22
Mean platelet volume fL	*8.2-11.9 U*		8	12.1		10.05		8	10.4	8	8.8	**0.12**	**1.14**		-	0.14	
Neutrophil count 10*9/L	*2-7.5U*		3.1	6.5	1.5	3.7	2.7	5.3	3.8	2.5	3.58	**0.03**	**1.03**		-0.71	0.42	-0.67
Lymphocyte count10*9/L	*1-4U*		2.2	1.2	1.4	1.6	1.4	1.7	1.8	1.7	1.65	**-0.03**	**0.97**		0.23	-0.5	-0.21
Monocyte counte 10*9/L	*0.2-0.8U*		0.4	0.4	0.3	0.37	0.3	0.4	0.3	0.3	0.33	**0.11**	**1.13**		-	0.25	-
Eosinophile count 10*9/L	*0-0.4U*		0.3	0	0.1	0.13	0.1	0.2	0	0.1	0.1	**0.25**	**1.33**		0.33		-
Basophil count 10*9/L	*0-0.1 U*		0.1	0	0	0.03	0.1	0	0	0.9	0.25	**-6.5**	**0.13**		1		
**Metabolism**																	
Serum ferritin ug/L	*30-250U*		69	27	53	49.67	13.9	82	38	41	43.73	**0.12**	**1.14**		-0.19	-0.41	0.23
Erythrocyte sedimentation rate (ESR) mm/h	*0-20 U*		2	2		2		8	2	5	5	**-1.5**	**0.4**		-3	-	
Serum C reactive protein mg/L	*0.5.oU*		1	<1.0	<1.0	1	<5.0	<1	<1.0	<1.0							
Serum total 25-hydroxy vitamin D level nmol/L	*50-200*		79	69	60	69.33	88		N/A	106	97	**-0.4**	**0.71**		1		-0.77
Serum calcium nmol/L			2.45	2.43	2.37	2.42	2.37	2.33	2.29	2.38	2.34	**0.03**	**1.03**		0.05	0.06	0
Serum adjusted calcium concentration nmol/L	*2.20-2.60 U*		2.39	2.34	2.33	2.35	2.32	2.35	2.33	2.42	2.36	**0**	**1**		0.02	0	-0.04
Serum total protein g/L	*60-80U*		69	79	70	72.67	68	63	68	68	66.75	**0.08**	**1.09**		0.09	0.14	0.03
Serum cholesterol mmol/L			4.9	5.8		5.35	6.1	5.3	5	5.9	5.58	**-0.04**	**0.96**		-0.08	0.14	
Serum HDL cholesterol nmol/l	*1.20-5.00U*		1.72	2.16		1.94	1.7	1.88	1.76	1.87	1.8	**0.07**	**1.08**		-0.09	0.19	
Serum cholesterol/ HDL ratio			2.8	2.7		2.75	3.6	2.8	2.8	3.2	3.1	**-0.13**	**0.89**		-	-0.04	
Serum non high density lipoprotein cholesterol mmol/l			3.2	3.6		3.4	4.4	3.4	3.2	4	3.75	**-0.1**	**0.91**		-0.06	0.11	
Triglyceride mmol/L			0.45			0.45	1.6	1.75		1.89	1.75	**-2.88**	**0.26**		-2.89		
Calculated LDL cholesterol level mmol/l			3			3		2.6		3.2	2.9	**0.03**	**1.03**		0.13		
Plasma glucose level mmol/L	*3.3-6.0U*		3.9	N/A	3	3.45			3.7		3.7	**-0.07**	**0.93**		1		1
Haemoglobin A1c level mmol/mol			37	34	37	36		36	37	38	37	**-0.03**	**0.97**		0.03	-0.09	-0.03
**Electrolytes, kidney and liver functions**																	
Serum inorganic phosphate mmol/l	*0.80-1.50U*		0.82	1.04	0.97	0.94		1.08	1.08	1.03	1.06	**-0.13**	**0.89**		-0.32	-0.04	-0.06
Serum urate level umol/L	*140-360U*		292	278	225	265	284	292	279	247	275.5	**-0.04**	**0.96**		-	0	-0.1
Serum sodium mmol/L	*133-146U*		138	141	142	140.3	137	139	143	139	139.5	**0.01**	**1.01**		-0.01	-0.01	0.02
Serum potassium mmol/L	*3.5-5.3U*		4.6	3.8	4.6	4.33	N/A	4.2	4	4.5	4.23	**0.02**	**1.02**		0.09	-0.05	0.02
Serum urea level mmol/L	*2.5-7.8*		5	6.1	5.5	5.53	5.6	6	4.6	7.5	5.93	**-0.07**	**0.93**		-0.2	0.25	-0.36
Serum creatinine umol/L	*49-90*		92	75	69	78.67	80	78	70	61	72.25	**0.08**	**1.09**		0.15	0.07	0.12
eGFR using creatinine (CKD-EPI) per 1.73m2 mL/min	*90-120U*		71	>90	103	87		86	>90	118	102	**-0.17**	**0.85**		-0.21		-0.15
Chloride	*94-112*																
Carbon dioxide mEq/L	*21-32*																
Serum albumin g/L	*35-50U*		43	51	42	45.33	44	39	41	38	40.5	**0.11**	**1.12**		0.09	0.2	0.1
Serum globulin g/L	*21-37U*		26		28	27	24	24		30	26	**0.04**	**1.04**		0.08		-0.07
Serum total bilirubin umol/L	*0-20U*		4	7	7	6		8	9	8	8.33	**-0.39**	**0.72**		-1	-0.29	-0.14
Serum alkaline phosphatase u/L	*30-130U*		67	51	47	55	63	54	55	56	57	**-0.04**	**0.96**		0.19	-0.08	-0.19
Serum alanine aminotransferase u/L	*0-55*		30	23	20	24.33		28	23	22	24.33	**-**	**1**		0.07	-	-0.1
Serum creatine kinase u/L	*25-200*		575	98	164	279	70	104	67	48	72.25	**0.74**	**3.86**		0.82	0.32	0.71
**Hormones**																	
Serum FSH level iu/L	*cycle >26.7*		5.7	6.3	6.4	6.13	5.6	3.2	3.1	1.1	3.25	**0.47**	**1.89**		0.44	0.51	0.83
Serum LH level iu/L	*cycle*		6.9	12.9	7.6	9.13	2.7	5.1	12.3	1.5	5.4	**0.41**	**1.69**		0.26	0.05	0.8
Oestradiol pmol/L	*cycle*		128	135	80	114.3	277	292	678	760	501.8	**-3.39**	**0.23**		-1.28	-4.02	-8.5
LH:FSH ratio			1.2		1.2	1.2		1.6		1.4	1.5	**-0.25**	**0.8**		-0.33		-0.17
Serum sex hormone binding globulin level nmol/l	*11.7-137.2U*		51.1	34.1	60	48.4		54.5	29.5	68	50.67	**-0.05**	**0.96**		-0.07	0.13	-0.13
Free androgen index %	*0.7-8.7U*		0.4	4.7	1.3	2.13		0.4	4.7	0.4	1.83	**0.14**	**1.16**		-	-	0.69
Serum TSH level miu/L	*0.35-4.94U*		1.39	1	2.13	1.51	2.84	1.02	1.24	1.42	1.63	**-0.08**	**0.92**		0.27	-0.24	0.33
Serum free T4 level pmol/L	*11-22U*		15.5		16	15.75		13		10.8	11.9	**0.24**	**1.32**		0.16		0.33
Serum vitamin B12 ng/L	*200-900U*		690	420	434	514.7	416	431	336	339	380.5	**0.26**	**1.35**		0.38	0.2	0.22
Serum folate ug/L	*3.0-20.5*		6.4	8.1	9.3	7.93	9.6	5.4	7	4.5	6.63	**0.16**	**1.2**		0.16	0.14	0.52
Serum progesterone nmol/L			<1	<1.6				18	<1.6	19							
Serum testosterone nmol/L	*0.5-1.9U*		0.2	1.6	0.8	0.87	0.7	0.2	1.4	0.3	0.65	**0.25**	**1.33**		-	0.13	0.63
Plasma prolactin level miu/L	*109-557U*		158	397	277	277.3	306	361		209	292	**-0.05**	**0.95**		-1.28	1	0.25
Serum cortisol nmol/L			250	392	280	307.3	583	203	556	105	361.8	**-0.18**	**0.85**		0.19	-0.42	0.63
